# Supplementary figures and images for: Fatty liver index as a predictive marker for the development of diabetes: A retrospective cohort study using Japanese health check-up data
Source: PLoS One. 2021 Sep 20;16(9):e0257352. doi: 10.1371/journal.pone.0257352 (PMC8451989; doi:10.1371/journal.pone.0257352)

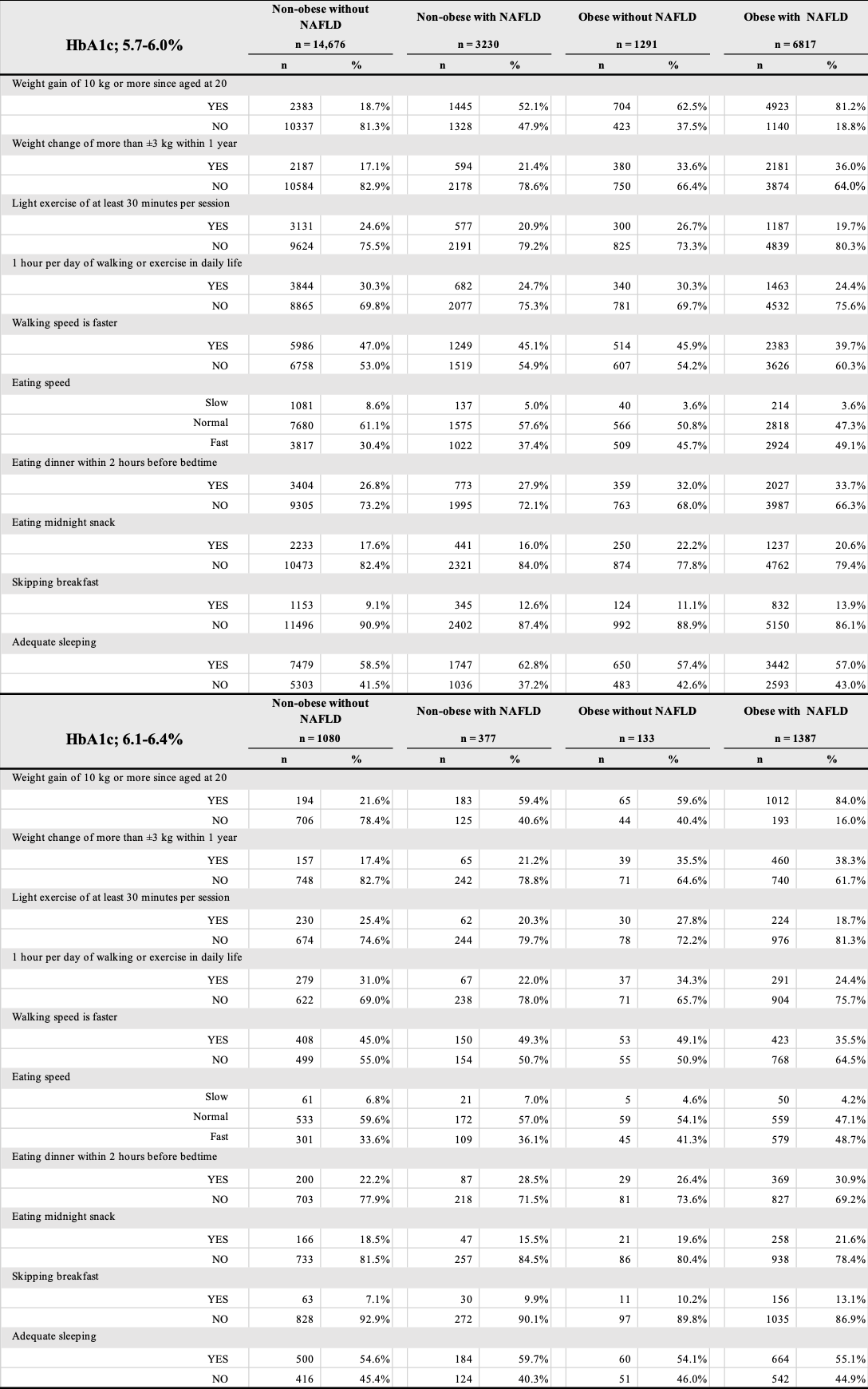

Supplement: S1 Table — (TIF) [file pone.0257352.s001.tif]

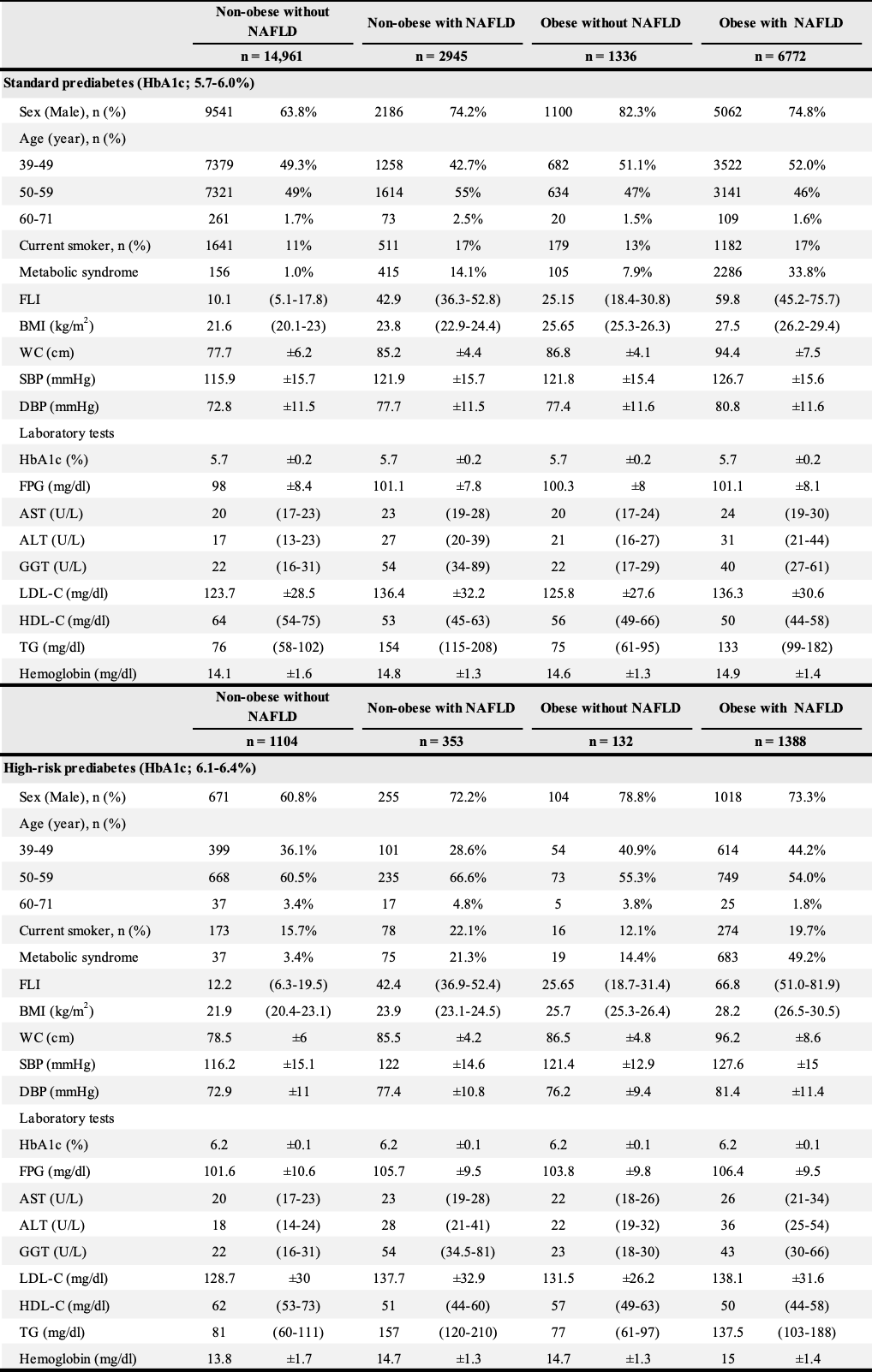

Supplement: S2 Table — (TIF) [file pone.0257352.s002.tif]
