# Supplementary material for: Fatty liver index as a predictive marker for the development of diabetes: A retrospective cohort study using Japanese health check-up data
Source: PLoS One. 2021 Sep 20;16(9):e0257352. doi: 10.1371/journal.pone.0257352 (PMC8451989; doi:10.1371/journal.pone.0257352)
Supplement: Attachment — Submitted filename: Response to Reviewers.docx [file pone.0257352.s003.docx]

June 30, 2021

Dr. Ming-Lung Yu, MD, PhD

Academic Editor

*PLoS ONE*

U.S. Headquarters Public Library of Science

1265 Battery Street Suite 200

San Francisco, CA 94111, USA

Re: Manuscript ID: PONE-D-21-12023

Dear Dr. Ming-Lung Yu:

Thank you for your e-mail and review of the manuscript titled “Fatty liver index as a predictive marker for the development of diabetes: a retrospective cohort study using Japanese health check-up data,” (PONE-D-21-12023) that we had submitted on April 21, 2021.

We have carried out additional sensitivity analyses of the sex-specific cutoff FLI value for NAFLD and the dose-dependent relationship between FLI and incidence of DM.

In addition, to avoid unsubstantiated claims, the conclusion has been revised as follows: The assessment of FLI combined with obesity revealed an association between FLI and a high risk of developing diabetes in a middle-aged Japanese population. To validate these results, a screening tool should be developed to effectively identify people at a high risk of developing diabetes from the population (particularly non-obese prediabetes) at a low health risk and unlikely to be targeted for health guidance.

Financial disclosure: We received no specific funding for this work.

The co-author, Shotaro Maeda, is an employee of KYORIN Pharmaceutical Co., but is also a researcher at the Teikyo University Graduate School of Public Health. He contributed to this study independently from KYORIN Pharmaceutical Co. Therefore, we have removed KYORIN Pharmaceutical Co. from affiliation in the manuscript.

Reply to Reviewer #1

We appreciate your comments on our manuscript. The comments have helped us significantly improve the paper. In particular, we acknowledge your valuable comments on the dose-dependent relationship between FLI and the development of diabetes and the sex-specific cutoff FLI value for NAFLD.

1. In this study, obesity was defined as body mass index (BMI) ≥25 kg/m2. However, the cutoff for obesity is 23.0 kg/m2 in Asia-Pacific countries, including Japan. The authors may perform subgroups analysis by BMI <23 versus ≥23 kg/m2.

Reply. The International Obesity Task Force recommended lower cutoffs of BMI ≥ 23 kg/m^2^ for overweight and ≥ 25.0 kg/m^2^ for obese Asian people, according to risks for type 2 diabetes and hypertension [32]. According to the Japanese guidelines, obesity is defined as BMI ≥ 25 kg/m^2^ [33]. Therefore, in the present study, obesity was defined as BMI ≥ 25 kg/m^2^. Because of insufficient explanation, we have revised the main text on page 9, lines 137–140 and added references #32 and #33.

2. The authors should provide the data that how many patients have metabolic syndrome. The correlation between and the development of DM should be taken into consideration.

Reply. The prevalence of metabolic syndrome according to Japanese diagnostic criteria [36] has been added to the patient background in Table 1, row 7. In the statistical analysis, all components of metabolic syndrome have been included in the model as components of FLI and other covariates. We could not add metabolic syndrome itself as a covariate because of multicollinearity.

3. In this study, the cutoff value for FLI in NAFLD was set at 30. The authors should analyze the dose-dependent relationship between FLI and incidence of DM.

Reply. FLI scores were transformed into seven levels of categorical variables, and the logistic regression analysis was performed for the development of diabetes. The results of the odds ratio are shown in Figure 4. There was a dose-dependent relationship between FLI and the development of diabetes in both standard and high-risk prediabetes that was more marked in standard prediabetes. These results have been added to page 20 lines 258–264 of the main text as a sub-analysis.

4. The cutoff value for FLI in NAFLD were different between male and female gender. Usually, the cut-off value was lower in females than in males. Regardless of gender, the cutoff value for FLI in NAFLD was set at 30 in this study. The authors should explain this important issue.

Reply. We have cited two references (#30 and #31) as validation studies in Asia of FLI in NAFLD diagnosed based on ultrasonography on page 9 lines 135–137 of the main text. One was a study in China with a cutoff value of 30 [30], and the other was a study in Taiwan with a cutoff value of 35 in men and 20 in women [31]. There is no absolute standard for the cutoff FLI value, which varies across populations. Movahedian et al. conducted a systematic review of 13 studies on FLI and the risk of diabetes incidence [7], wherein the cutoffs varied across studies. Of the 12 articles that included both men and women, only one by Hirata et al. separated the cutoff value by sex [10]. In the present study, FLI could not be validated for NAFLD because ultrasonography was not performed. The cutoff FLI value for both men and women was set at 30, but for the sensitivity analysis, it was set at 35 for men and 20 for women, which were also analyzed and have been added to Supplementary Table 2 and page 19 lines 254–257 of the main text. The results were not remarkably changed, but one difference was that in high-risk prediabetes, the non-obese FLI-NAFLD group had a significant OR ranging from 1.42 (0.99–2.03) to 1.68 (1.18–2.39).

5. The inclusion criteria (8) did not have normoglycemia (HbA1c ≥6.5% or FPG ≥126 mg/dL or use of antidiabetics) at FY2015. Patients with normoglycemia should have HbA1c < 5.7% or FPG <100 mg/dL. Please correct.

Reply. We corrected it as follows: normoglycemia (HbA1c <5.7% and FPG <100 mg/dL).

Reply to Reviewer #2:

We appreciate your insightful comments on our manuscript. The comments have helped us significantly improve the paper. In particular, we acknowledge your valuable comment on the sex ratio of FLI-NAFLD prevalence and that of the risk of developing diabetes.

Major consideration:
Table 1: the gender ratio of non-obese with FLI-NAFLD and obese with FLI-NAFLD were almost male (90.4% and 79.8%), why?

Reply. This study included 19,937 (69%) men and 9,054 (XX%) women. In addition, the prevalence of NAFLD, defined as FLI ≥ 30, was 49% (9,748) in men and 23% (2,063) in women. These differences in prevalence could have affected the sex ratio in FLI-NAFLD. FLI values were significantly higher in men than in women, which seemed to cause a sex difference in the prevalence of FLI-NAFLD. These findings have been added to page 23 lines 312–316 of the main text.

Minor consideration:

1. Table 1, current smoker: is the smokers almost male? What is the proportion?

Reply. Current smokers included 93.5% men and 6.5% women. In addition, the current smoking rate by sex was 19% in men and 2.9% in women.

2. Table 2: the male risk is 1.99 in univariate model, and 0.76 in multivariate model, can you explain it?

Reply. For the risk in men, there seemed to be a strong confounding effect of FLI, laboratory values (particularly SBP, FPG, and HDL), and lifestyle (particularly current smoking). After adjusting for these confounding factors, women were at a higher risk of developing diabetes compared to men. These findings have been added to page 23 lines 322–326 of the main text.

3. How to define walking and eating speed is faster?

Reply. In Japan, all insured people over 40 years of age are required to undergo a specific medical checkup. In the specific medical checkup, there was a standardized self-administered questionnaire, and results of answers to the questionnaire were used. The questions were as follows: (1) Is your walking speed faster than that of a person of the same age? (2) How fast do you eat compared to other people?

4. Page 7, line 116: (3) had no outlier data: how to define “outlier” data?

Reply. The order of the flowchart of the analysis participants in Figure 1 has been modified for clarity. The final analysis participants were unchanged. The exclusion of outliers was the third from the end, before the exclusion of diabetic and normoglycemic subjects. Outlier exclusion was performed manually, and eight patients were excluded. Outliers were those with an HbA1c of 0% or 63% and a WC of 28.6 cm or less in FY2015 and those with an HbA1c of 0% or 58% in FY2018. We believe that these outliers were due to data entry errors.

Figures and Table:

To improve the manuscript according to reviewers’ comments, we have changed the figure composition as follows:

Figure 1 has been revised to reflect a change in the order of the flowchart of the analysis participants.

A new figure has been inserted as Figure 4.

Table S2 has been inserted in the manuscript.

We believe that we have addressed the reviewers’ comments and hope that the revised manuscript is now acceptable for publication in *PLoS ONE*. Thank you for your consideration.

Sincerely yours,

Atsushi Kitazawa, MD

Teikyo University Graduate School of Public Health,

Tokyo, Japan

[Postal address] 1738605

[Phone number] 81-3-3964-1211

[Fax number] 81-3-3964-1058

[Email address] [sph-akitazawa@med.teikyo-u.ac.jp](about:blank)
