# Supplementary material for: Fatty liver index as a predictive marker for the development of diabetes: A retrospective cohort study using Japanese health check-up data
Source: PLoS One. 2021 Sep 20;16(9):e0257352. doi: 10.1371/journal.pone.0257352 (PMC8451989; doi:10.1371/journal.pone.0257352)
Supplement: Attachment — Submitted filename: Response to Reviewers.docx [file pone.0257352.s004.docx]

August 5, 2021

Dr. Ming-Lung Yu, MD, PhD

Academic Editor

*PLoS ONE*

U.S. Headquarters Public Library of Science

1265 Battery Street Suite 200

San Francisco, CA 94111, USA

Re: Manuscript ID: PONE-D-21-12023

Dear Dr. Ming-Lung Yu:

Thank you for your e-mail and second review of the manuscript titled “Fatty liver index as a predictive marker for the development of diabetes: a retrospective cohort study using Japanese health check-up data,” (PONE-D-21-12023) that we had submitted on April 21, 2021.

We have added the results of sensitivity analysis with a cutoff value of FLI of 35 for males and 20 for females are shown in Table 4, and descriptive analyses for baseline characteristics of the eligible subjects are shown in Table in S2 Table.

Reply to Reviewer #1

We appreciate your comments on our manuscript.

Reply to Reviewer #2

**1.According to author's reply, you use a new cutoff value of FLI of 35 for males and 20 for females and it seems have better results in this study. "the odds ratio of non-obese FLI-NAFLD in high-risk prediabetes changed from 1.42 (0.99-2.03) to 1.68 (1.18-2.39), which was significant. Can you upload these differences in fully supplement tables and why don't you use the new cutoff value instead of the original one?**

Reply.

First, because the protocol was initially determined with FLI 30 as the cutoff value. Secondly, there is no common consensus on the cutoff value of FLI in Asia. Therefore, we thought it should be easier to use the FLI as a screening tool in health examination results. Therefore, we used a common cutoff value for men and women.

The results of sensitivity analysis with a cutoff value of FLI of 35 for males and 20 for females are shown in Table 4, and descriptive analyses for baseline characteristics of the eligible subjects are shown in Table in S2 Table.

**2.Why the original enrolled subjects are male dominant? 19,937 in male v.s. 9054 in female?**

Reply.

This is because the gender distribution of the employers in this insurance association is significantly more male.

Figures and Table:

To improve the manuscript according to reviewers’ comments, we have changed the figure composition as follows:

The results of sensitivity analysis with a cutoff value of FLI of 35 for males and 20 for females are shown in Table 4, and descriptive analyses for baseline characteristics of the eligible subjects are shown in Table in S2 Table.

We believe that we have addressed the reviewers’ comments and hope that the revised manuscript is now acceptable for publication in *PLoS ONE*. Thank you for your consideration.

Sincerely yours,

Atsushi Kitazawa, MD

Teikyo University Graduate School of Public Health,

Tokyo, Japan

[Postal address] 1738605

[Phone number] 81-3-3964-1211

[Fax number] 81-3-3964-1058

[Email address] [sph-akitazawa@med.teikyo-u.ac.jp](about:blank)
